# Training characteristics of male and female WorldTour professional road cyclists before the competitive phase

**DOI:** 10.5114/biolsport.2026.156234

**Published:** 2026-02-23

**Authors:** Manuel Mateo-March, David Barranco-Gil, Xabier Muriel, Jesús G. Pallarés, Pedro L. Valenzuela

**Affiliations:** 1 Department of Sport Sciences, Sports Research Center, Universidad Miguel Hernández de Elche, Alicante, Spain; 2 Department of Sports Sciences. Faculty of Medicine, Health and Sports, Universidad Europea de Madrid, Madrid, Spain; 3 Health, Physical Activity, and Sports Science Laboratory, Department of Physical Activity and Sports, Faculty of Education and Sport, University of Deusto, San Sebastián, Spain; 4 Human Performance and Sports Science Laboratory, Faculty of Sport Sciences, University of Murcia, Murcia, Spain; 5 GENUD Toledo Research Group, Faculty of Sport Sciences, University of Castilla-La Mancha, Toledo, Spain; 6 Department of Systems Biology, University of Alcalá, Madrid, Spain

**Keywords:** Training, Intensity, Performance, Cycling, Endurance, Sex

## Abstract

Evidence regarding the training characteristics of professional cyclists is limited, particularly for female athletes. We aimed to compare the week-by-week training characteristics of female and male professional road cyclists of the highest competitive level. We analysed data from 16 female (age 26 ± 5 years) and 16 male WorldTour cyclists (age 29 ± 6 years). Power output (PO) and heart rate (HR) were registered during the 10 weeks preceding the first competition of the season, and different measures of training load (e.g., total time, training stress score [TSS], training impulse [eTRIMP]) and training intensity distribution (i.e., time spent in each intensity zone) were determined. Female and male cyclists completed a similar number of training sessions (5.9 ± 0.9 vs 6.0 ± 0.9 sessions/week, respectively; p = 0.760), although the latter trained more hours (16.7 ± 2.6 vs 19.1 ± 2.7 hours/week; p = 0.016). A significant reduction of training volume was observed during the last week before the competitive phase, particularly in females (11.2 ± 4.6 vs 17.7 ± 4.9 hours; p < 0.001). Most cyclists (> 90%) followed a pyramidal training intensity distribution through the study period regardless of sex, although females spent less absolute and relative time in low-intensity zones measured by both PO (p < 0.001) and HR (p = 0.009), with more time in higher-intensity zones. No differences were found in relative training load indicators such as TSS (p = 0.986) or eTRIMP (p = 0.612) during the study. Female cyclists show lower training volumes—particularly at low intensity—than male cyclists. However, similar relative training loads are found in both sexes, likely due to the higher relative training intensity of female cyclists.

## INTRODUCTION

Professional road cyclists are considered to represent the epitome of endurance exercise performance [1–3]. It is worth noting, however, that most research on the physical demands of road cycling has been conducted in male athletes [4, 5]. Although female cycling is growing in popularity, evidence on the physical demands experienced by professional female cyclists remains limited [6–9]. The lack of data in female cyclists is problematic in real-world contexts, as extrapolating findings from males risks suboptimal training prescriptions that overlook sex-specific physiological and race demands, potentially hindering performance and increasing injury risk.

To sustain such high physical demands, cyclists undergo strenuous training regimens, but, as highlighted in a recent systematic review, scarce evidence exists on the training characteristics (e.g., training volume, intensity distribution) of professional cyclists —particularly female ones— [10]. Some reports allow us to at least describe the most common training strategies applied by world-class professional cyclists, but evidence is mostly restricted to case studies in professional male cyclists [11–13]. Indeed, a recent systematic review found only one study reporting the training characteristics of professional female cyclists [14].

Specifically, to the best of our knowledge, only Van Erp et al. have reported the training characteristics of professional female cyclists [15]. These authors compared the average training characteristics of 20 male and 10 female professional road cyclists (competing at Pro-Continental and WorldTour level) collected during 1 to 4 years [15]. However, the authors analysed average data from the whole seasons (including the competitive phase), and therefore it is possible that the presence of competitions biased some of the measures. This is of particular relevance given that there are differences between male and female races, with the latter having a lower distance and riding time, but greater relative intensity [6, 7]. Moreover, the cycling season includes a higher number of races in males compared to females, which increases the bias. In addition, the progression of training characteristics of female and male cyclists up to the competition phase also remains unknown.

Under this context, the aim of the present study was to compare the week-by-week training characteristics of female and male professional road cyclists of the highest level (WorldTour) during the precompetition phase. We hypothesized that female cyclists would exhibit lower training volume but higher relative intensities compared to males, reflecting sex-specific race demands.

## MATERIALS AND METHODS

### Participants

The protocol complied with the Declaration of Helsinki. We used data from all male and female riders pertaining to one professional team of the same level (WorldTour) during the 2018–2019 season (although they had different coaches and could train differently). Data were collected from October to December 2018, preceding the 2019 season. Specifically, we analysed training data from the 10 weeks preceding the first competition of the season—which represented the entire pre-season build-up—in order to avoid the confounding effect of races in training characteristics. This timeframe was considered relevant as it captures the progressive accumulation and tapering of training loads specific to pre-competitive preparation without competitive interference. Finally, a total of 1696 training sessions (~50 sessions per cyclist) were obtained from 16 female (age 26 ± 5 years, experience in the professional category 6 ± 4 years, weight 65 ± 5 kg, height 167 ± 7 cm) and 16 male cyclists (age 29 ± 6 years, experience in the professional category 7 ± 5 years, weight 67 ± 6 kg, height 180 ± 5 cm). Eligible participants were all cyclists competing in the team. No specific eligibility criteria were set, but cyclists who experienced injuries or illnesses during this 10-week period and thus did not complete all training weeks were excluded from the analyses. As descriptive variables, we estimated participants’ highest critical power (CP) through the 1/time method using the 2-, 5-, and 12-min mean maximal performances (MMPs), as these durations provide a robust fit for the power-duration relationship in field conditions [16]; and maximum oxygen uptake (${\dot{\text{V}}\text{O}}_{2\max}$) from the highest 5-min MMP recorded during the study period, regardless of prior accumulated work, using the method described by Borszcz et al. (15).

### Measures

Power output (PO) was registered during all training sessions (Shimano Dura-Ace FCRC9100-P, Shimano, Sakia, Japan; and Power2Max GmbH, Owingen, Germany). A zero-offset was performed according to manufacturers’ instructions before each training session, and PO data were visually checked for potential spikes and manually corrected when necessary, as explained elsewhere [17, 18]. Heart rate (HR) was also analysed through a HR chest strap (Garmin HRM Dual Heart Rate Chest Strap, Kansas City, MO).

In order to be included in the analyses, cyclists were required to have registered the PO or HR of at least 80% of the total training time of each week. Eventually we had available PO data for 15.3 ± 2.6 and 17.8 ± 2.5 hours per week for females and males, respectively (92% and 93% of the total training time, respectively), and available HR data for 14.4 ± 3.1 and 16.5 ± 3.0 hours per week, respectively (82% and 87% of the total training time, respectively).

### Training load indicators

The metrics used in the present study were registered as explained elsewhere [6]. Briefly, these included total time, mechanical work (kJ), and derived load scores. In addition to the total training time, from PO data we computed the kJ spent (mechanical energy) and the Training Stress Score (TSS), which was computed using the following equation:

TSS=((t×NP×IF)/(FTP×3600))×100

Where t is the duration of the stage in seconds, NP is the normalized power attained during the stage, and IF (intensity factor) is the ratio between the NP and the functional threshold power (FTP) [19]. For this purpose, the FTP of each week was obtained from the highest 20-minute PO attained during the preceding 4 weeks to account for potential fitness adaptations within the 10-week pre-competitive phase.

Using HR data, we also computed Edwards’ training impulse (TRIMP) based on the time spent in the five pre-defined HR zones, as explained elsewhere [15]. Briefly, HR zones were computed based on the following *intensity zones*: 1 (≤ 49% of maximum HR (HRmax)), 2 (50% to 59% of HRmax), 3 (60% to 69% of HRmax), 4 (70% to 79% of HRmax), 5 (80% to 89% of HRmax), and 6 (≥ 90% of HRmax). Then, TRIMP were computed by multiplying the time spent in each zone by a zone-specific arbitrary weighting factor (HR zone 1–2: weighting factor = 1, HR zone 3: weighting factor = 2, HR zone 4: weighting factor = 3, HR zone 5: weighting factor = 4, HR zone 6: weighting factor = 5) and then summated to provide a total TRIMP score.

### Training intensity distribution

Training intensity distribution (TID) was quantified through assessing the time spent in 3 different training zones: low-intensity (LIT, zone 1), moderate-intensity (MIT, zone 2) and high-intensity (HIT, zone 3) training [20, 21].

For the determination of training zone based on PO, we used the estimated CP to separate MIT and HIT, which is considered a valid indicator of the transition from the heavy to the severe intensity domain [22]. For this purpose, we determined CP from training data as explained elsewhere [18], and updated this value each 4 weeks. As we could not directly measure the ventilatory threshold or any other physiological surrogate, taking into account a recent meta-analysis, the boundary between LIT and MIT was assumed to be 70% of CP [23]. For the definition of training zones based on HR, following the procedures explained elsewhere, LIT ranged from 60 to 82% of HRmax, MIT ranged from 82 to 88%, and HIT ranged from 89 to 100% [24].

Different types of TID were determined considering the proportion of time spent in LIT, MIT and HIT, as explained elsewhere [20]. A pyramidal TID was considered when %LIT was greater than %MIT and %MIT was greater than %HIT. A threshold TID was considered when %MIT was greater than both %LIT and %HIT. A high-intensity TID was considered if %HIT was greater than both %LIT and %MIT. Finally, a polarized TID was considered when %LIT was greater than %MIT, but %HIT was greater than %MIT. To confirm the type of TID, we also computed a polarization index from the proportion of time spent in each intensity zone, using the formula proposed elsewhere [20]:

Polarization index(a.u.)=log10(LIT/MIT×HIT×100).

Where LIT, MIT and HIT represent the fraction (given percentage/100) of the time spent in that zone. A polarized index > 2.00 a.u. was necessary to confirm a polarized TID.

### Statistical analyses

Data are shown as mean ± standard deviation (SD). Overall differences between *male* and *female* cyclists were assessed using unpaired Student’s t-tests. Due to the presence of missing data in some weeks in some participants (< 10% of total data), a linear mixed-effects model (LMM) was used to examine the effect of time and sex on each variable. The model included both time (week number) and sex as categorical variables, and their interaction was incorporated as a fixed effect. A random intercept and slope for time were included at the individual level to account for within-subject correlation. The covariance structure was specified as unstructured to allow for maximum flexibility in modelling correlations across repeated measurements. Post hoc comparisons were performed using marginal means (margins) to assess differences between men and women at each week, with Bonferroni correction applied to adjust for multiple comparisons. The magnitude of the differences was also determined using effect sizes (ES, Cohen’s d). All analyses were conducted in Stata (13.0, TX: StataCorp LLC) with statistical significance set at p < 0.05.

## RESULTS

Male cyclists had a higher estimated CP (380 ± 34 vs 237 ± 24 W, respectively; p < 0.001) and ${\dot{\text{V}}\text{O}}_{2\max}$ (73.3 ± 3.7 vs 62.6 ± 3.8 ml/ kg/min, p < 0.001) compared to females, but no significant difference was found for HR_max_ (194 ± 6 vs 193 ± 12 bpm, p = 0.758).

## Training loads

Average measures of weekly training loads during the study period are shown in Table 1. Female and male cyclists completed a similar number of weekly training sessions (Δ = 1.7%; ES = 0.11, p = 0.760), but males trained for more hours (Δ = 12.6%; ES = 0.91, p = 0.016) and covered a greater distance (Δ = 20.0%; ES = 1.16, p = 0.003), with a greater elevation gain (Δ = 18.2%; ES = 1.06, p = 0.006). Moreover, male cyclists attained a greater quantity of mechanical work (Δ = 39.8%; ES = 2.37, p < 0.001), but no significant differences were found in relative indicators of training load such as TSS (Δ = 0.1%; ES = 0.01, p = 0.986) or eTRIMP (Δ = 3.6%; ES = 0.19, p = 0.612).

**TABLE 1:** Overall weekly training loads during the 10-week period in female and male professional cyclists.

	Female (n=16)	Male (n=16)	p-value
Training sessions (n per week)	5.9 ± 0.9	6.0 ± 0.9	0.760
Total training volume (hours per week)	16.7 ± 2.6	19.1 ± 2.7	0.016
Distance (km per week)	428 ± 108	535 ± 76	0.003
Elevation gain (m per week)	5,602 ± 1,058	6,845 ± 1,319	0.006
eTRIMP (a.u. per week)	2,605 ± 643	2,701 ± 384	0.612
TSS (a.u. per week)	847 ± 169	848 ± 121	0.986
Work (kJ per week)	7,458 ± 1,639	12,397 ± 2,531	< 0.001

Abbreviations: TSS, training stress score; eTRIMP, Edwards’ Training Impulse.

**FIG. 1:**
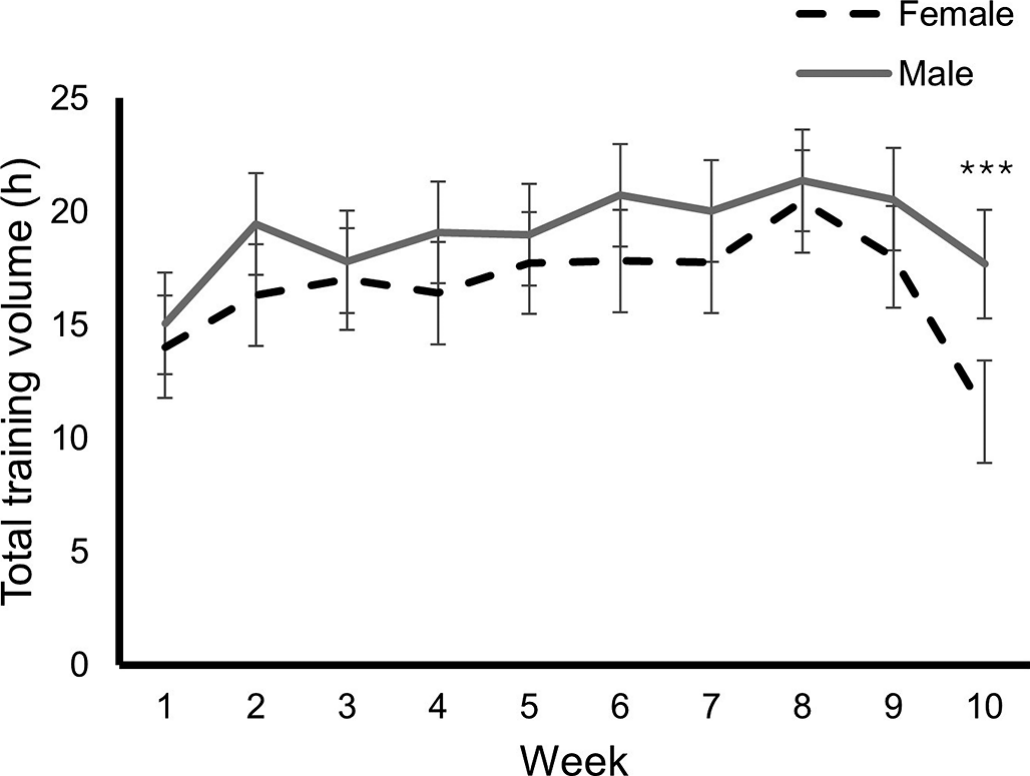
Weekly training volume (mean and 95% confidence interval) in female and male professional cyclists. Significant differences between sexes: ***p < 0.001.

There was no significant time (p = 0.739) or sex by time effect for training volume (p = 0.111), but a non-significant progressive increase in training volume was observed up to the second last week before the competitive phase, when a reduction in training volume was observed, which was of significantly greater magnitude in women (p < 0.001) (Figure 1). Specifically, training volume peaked at week 9 (females: 20.1 ± 3.2 h; males: 22.5 ± 2.8 h) before tapering to week 10 (females: 11.2 ± 4.6 h, -44%; males: 17.7 ± 4.9 h, -21%; p < 0.001).

## Training intensity distribution

**FIG. 2:**
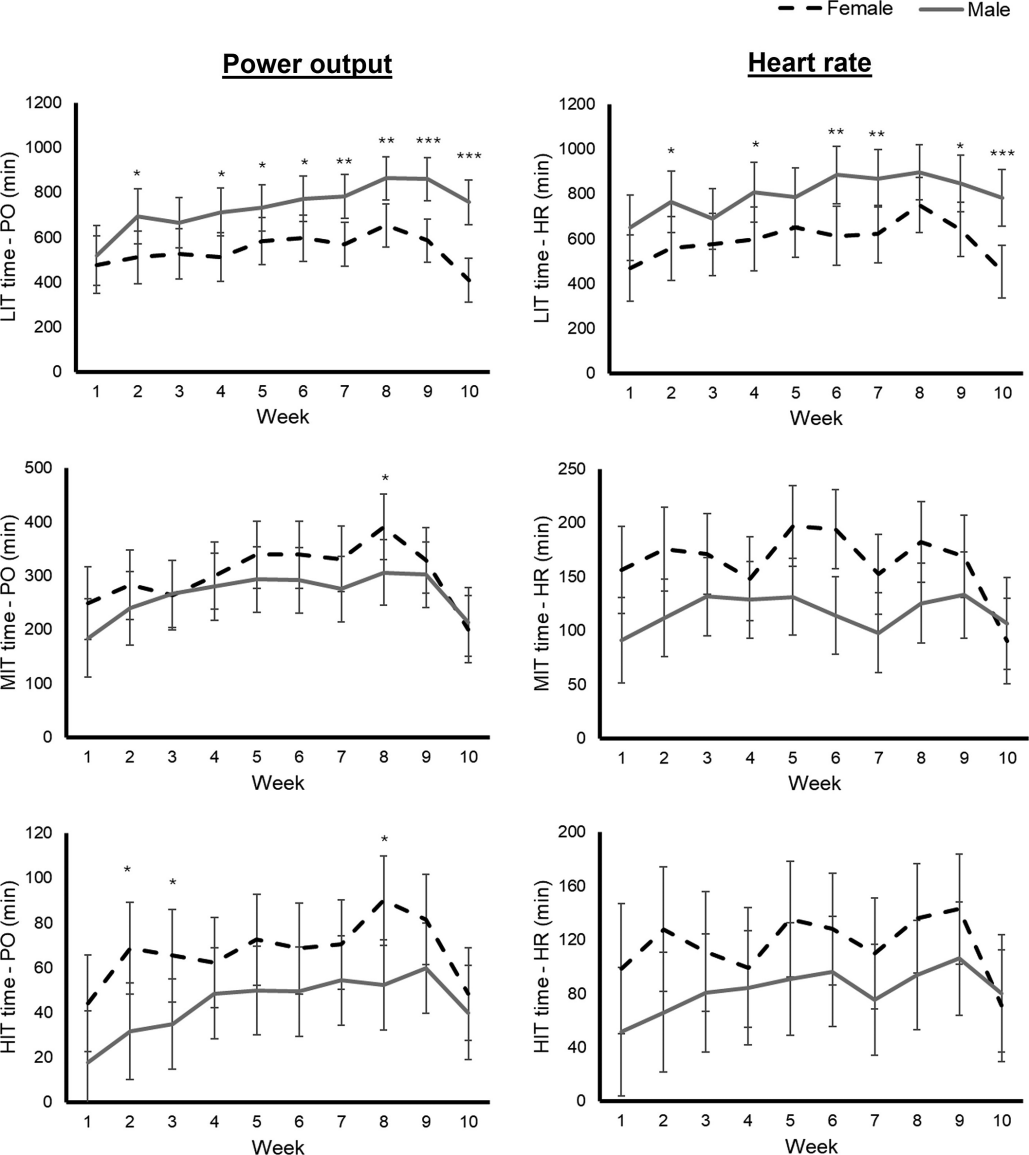
Training time (mean and 95% confidence interval) in each intensity zone considering power output (PO) and heart rate (HR). Abbreviations: LIT, low-intensity training; MIT, moderate-intensity training; HIT, high-intensity training. Significant differences between sexes: *p < 0.05, **p < 0.01, ***p < 0.001.

**FIG. 3:**
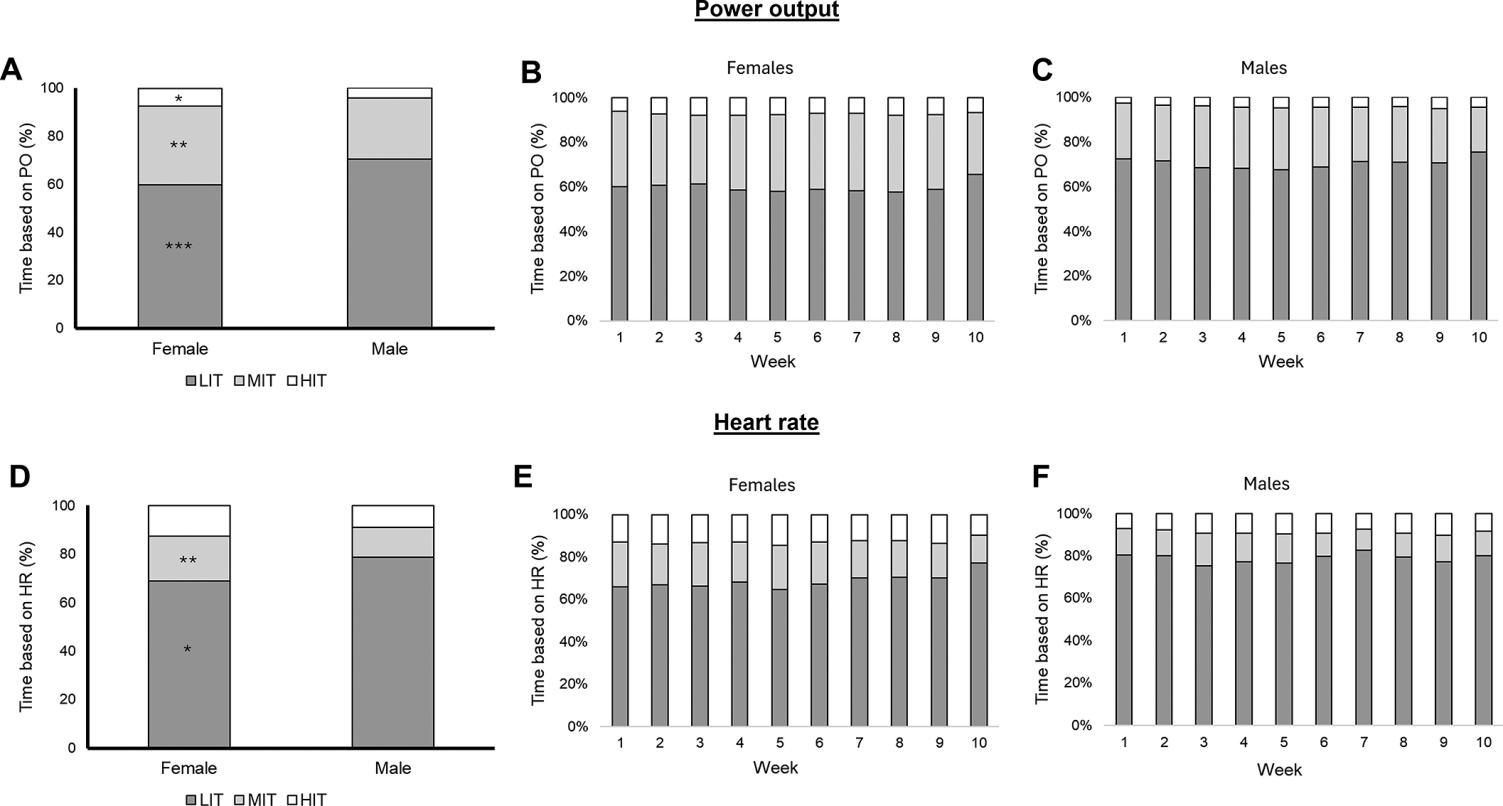
Average and weekly training intensity distribution in female and male cyclists considering power output (average in panel A, and weekly distribution in panels B and C) and heart rate (average in panel D, and weekly distribution in panels E and F). Abbreviations: LIT, low-intensity training; MIT, moderate-intensity training; HIT, high-intensity training. Significant differences between male and female cyclists for the time spent in that intensity zone: *p < 0.05, **p < 0.01, ***p < 0.001.

The absolute and relative amount of time spent in each intensity zone is shown in Table 2. In absolute units, male cyclists completed significantly more time in LIT when measured by either PO or HR, with significantly less time spent in HIT when measured by PO, and less time spent in MIT when considering HR (Table 2). The same trend was observed in weekly analyses (Figure 2). Of note, a reduction of the time in MIT and HIT was observed in the last two weeks, particularly in female cyclists (Figure 2). When expressed in relative units, male cyclists still showed a higher proportion of time in LIT, with a lower proportion of time spent in both MIT and HIT when measured by PO, and a lower proportion of MIT when measured by HR (Table 2, Figure 3). The proportion of time in each intensity zone remained relatively constant through the study period in both sexes (Figure 3).

**TABLE 2:** Overall training intensity distribution during the 10-week period in female and male professional cyclists.

	Female (n=16)	Male (n=16)	p-value
LIT power (min)	544 ± 156	750 ± 140	< 0.001
MIT power (min)	305 ± 98	270 ± 60	0.238
HIT power (min)	68 ± 36	45 ± 21	0.037
LIT power (%)	59.7 ± 10.4	70.4 ± 6.2	0.001
MIT power (%)	32.8 ± 9.0	25.4 ± 5.1	0.008
HIT power (%)	7.4 ± 3.8	4.2 ± 1.8	0.005
Polarized index power	1.07 ± 0.25	1.03 ± 0.15	0.559
LIT HR (min)	589 ± 198	791 ± 208	0.009
MIT HR (min)	160 ± 62	117 ± 29	0.020
HIT HR (min)	114 ± 97	84 ± 38	0.249
LIT HR (%)	69.0 ± 15.3	78.8 ± 8.6	0.033
MIT HR (%)	18.3 ± 6.3	12.4 ± 4.6	0.005
HIT HR (%)	12.8 ± 9.7	8.8 ± 4.8	0.159
Polarized index HR	1.54 ± 0.24	1.71 ± 0.14	0.020

Abbreviations: LIT, low-intensity training; MIT, moderate-intensity training; HIT, high-intensity training; HR, heart rate. Note: Polarized index values < 2 confirm pyramidal TID for all cyclists; no polarized distributions were observed.

When measured by PO, all cyclists followed a pyramidal TID except for one female cyclist who followed a threshold TID (43.4%, 53.4 and 3.2% of time in LIT, MIT and HIT). When measured by HR, most cyclists still had a pyramidal TID, although in 2 female cyclists and 3 male cyclists the proportion of time spent at HIT was slightly greater than the proportion of time spent at MIT (although only reaching the polarized index threshold of ≥ 2 in one case).

## DISCUSSION

The main finding of the present study was that professional male road cyclists complete a higher total training volume (Table 1), particularly due to more absolute and relative time in low-intensity zones – even if they spend less time in higher-intensity zones (Table 2). Despite the lower training volume of females, no differences were found for relative training load indicators, likely due to their higher relative training intensity. These differences remained overall constant through the whole study period, albeit a particularly lower training volume was observed in females before the competitive phase.

In the present study, both female and male professional cyclists accumulated a large training volume (> 15 and 19 hours/week on average). While experimental evidence (e.g., randomized controlled trials) is warranted to confirm the actual role of training load/volume on performance in professional cyclists, previous observational studies overall support the importance of training volume for endurance and particularly cycling performance. Case series reveal that professional male cyclists who attained a top position in Grand Tours such as Giro d’Italia trained > 15 hours/week on average, with some weeks reaching > 30 hours [11–13]. An observational study by Sanders et al. reported that higher weekly training loads were positively associated with performance indicators (e.g., lactate threshold) in competitive male cyclists [25]. Similarly, Leo et al. reported that U23 male cyclists who eventually transitioned to international/ elite ranks trained more hours and accumulated a greater mechanical work than their counterparts who did not transition [26]. Thus, attaining a high training volume might be a cornerstone for cycling performance. It should be noted, nonetheless, that we observed a higher training volume in male compared to female cyclists (Table 1, Figure 1). Evidence on the training loads of professional female cyclists is scarce, as highlighted in a recent systematic review [14]. However, in line with our findings, Van Erp et al. also reported a lower training volume in female cyclists (142 vs 185 minutes per session) [15]. These sex differences could also be a consequence of the specific demands of competitions, as male races are characterized by longer riding distance and time compared to female ones [6], necessitating greater endurance-building volume in males.

In the present study we also analysed the training intensity distribution of male and female professional cyclists, finding that most of them adopted a pyramidal distribution (Figure 3). Experimental evidence on the optimal training intensity distribution in professional cyclists is lacking, but, in line with our findings, evidence suggests that most elite endurance athletes adopt a pyramidal intensity distribution, with a large volume performed at low intensity [27]. For instance, Sperlich et al. reported that cyclists spend ~65% of the time in Z1, ~29% in Z2, and ~6% in Z3 [28]. However, the training intensity distribution of female cyclists remains largely unstudied. In this regard, we found that female cyclists spend less absolute and relative time in low-intensity zones, with higher relative time at moderate intensity. Indeed, due to the higher relative training intensity of female cyclists, we observed no differences in relative training load indicators such as Training Stress Score (TSS) or Edwards’ Training Impulse (eTRIMP) despite the abovementioned differences in training volume (Table 1). Similarly to our results, Van Erp et al. reported that female cyclists spent less absolute and relative time in low-intensity zones (particularly when measured by heart rate) but more time at higher intensities. Although speculative, the higher MIT in females may enhance glycolytic capacity, aligning with race demands [6, 9]. This result could be important due to the potential role of accumulating large training volume at low intensity, as suggested by observational studies in endurance athletes [29]. Moreover, in the present study the greater amount of time spent at moderate intensities in female cyclists led to a reduced polarized index. In this regard, some evidence suggests that a polarized training intensity distribution could lead to larger improvements in fitness indicators (i.e., maximal oxygen uptake) in competitive athletes [30], although experimental evidence is needed to determine the optimal training intensity distribution for each sex. Notwithstanding, these differences in training intensity distribution might be partly explained by the specific characteristics of female races, as Sanders et al. reported that female cyclists spend a greater proportion of time in highintensity HR zones, and that women’s races are associated with higher training load indicators when expressed relative to distance (e.g., TSS or TRIMP per km) [6].

It is also worth noting that both male and female cyclists tended to reduce their training load in the last week before the competitive phase, which is consistent with the habitual strategy followed by endurance athletes [31]. However, although both male and female cyclists reduced their training volume, which has proven to be an effective tapering strategy [32], this reduction was markedly greater in females. Moreover, although it has been recommended that during tapering athletes should maintain training intensity despite reducing training volume [31, 32], in the present study female cyclists showed a marked reduction of training volume specifically at moderate and high intensity, which would likely reduce the potential residual fatigue when starting the first races, but could also reduce performance if the tapering phase was too long. Further research is warranted to confirm the best tapering strategy in male and female cyclists. Particularly, the observed intensity reduction in females warrants caution, as meta-analytical evidence suggests that maintaining intensity preserves performance gains [32].

Despite their observational nature, the present findings might reinforce the importance of training volume for cycling performance, with even larger training volumes observed in male cyclists (> 15 and 19 hours/week on average for male and female cyclists). Moreover, our results show that cyclists spend most of the training time at low intensity regardless of sex, although females show a higher proportion of time at moderate-to-high intensities. The sex-specific patterns identified here may reflect adaptations to the distinct competitive demands faced by male and female cyclists [6]. Female races, often shorter in duration yet higher in relative intensity, likely contribute to the increased emphasis on moderate-to-high intensity training among female athletes. In contrast, the longer distances of male races may necessitate greater low-intensity volume to build endurance capacity. These insights highlight the need for tailored training approaches that account for the unique physiological and competitive profiles of each sex. Further evidence is however warranted to confirm the optimal training volume/load and intensity distribution in male and female cyclists.

Some limitations of the present study should be acknowledged. Due to the observational nature of our study, we cannot draw conclusions on cause-effect associations. Moreover, we did not monitor physical fitness through ad hoc tests, and therefore we could not analyse whether some specific training characteristics were associated with improvements on physical fitness. Moreover, there was a lack of control of contextual factors (e.g., team strategies, recovery, nutrition), which may introduce potential confounding. In line with this, we estimated CP (and training intensity zones) based on field-based training data, excluding the competitive period to reduce the bias that this might introduce in training intensity distribution. Although this procedure has proven valid overall [18], we might have underestimated the actual CP, leading to an overestimation of the time spent in high-intensity zones. Additionally, relative training load metrics like TSS are susceptible to individual errors in FTP estimation, and they might not sufficiently account for the influence of intensity with respect to volume. Finally, we did not monitor the number of strength training or other off-the-bike sessions, which can also affect overall training loads. The use of multiple power meter models may have also introduced minor measurement variability. In turn, despite the limited sample size, the participants represent the highest competitive level (UCI WorldTour), providing a meaningful training reference for lower-level categories (UCI Continental, U23 or even juniors). The continuous monitoring of week-by-week training characteristics of professional male and female professional cyclists using both PO and HR should also be considered a major strength.

## CONCLUSIONS

Professional male road cyclists complete a higher total training volume compared to female ones, mostly due to more time in low-intensity zones. Moreover, although most cyclists followed a pyramidal training intensity distribution regardless of sex, female cyclists spent a lower proportion of time at low intensity and a higher proportion at moderate-to-high intensity. Thus, no differences were found for relative training load indicators. These sex-specific patterns suggest that training prescriptions prioritize volume in males and relative intensity in females. Prospective studies and particularly randomized controlled trials are needed to elucidate the best training strategy for each sex.
